# The prognostic value of national early warning scores (NEWS) during transfer of care from community settings to hospital: a retrospective service evaluation

**DOI:** 10.3399/bjgpopen20X101071

**Published:** 2020-05-13

**Authors:** Matthew Inada-Kim, Thomas Knight, Michelle Sullivan, Mark Ainsworth-Smith, Neil Pike, Mathew Richardson, Gail Hayward, Daniel Lasserson

**Affiliations:** 1 Hampshire Hospitals NHS Foundation Trust, Winchester, UK; 2 Institute of Applied Health Research, College of Medical and Dental Sciences, University of Birmingham, Birmingham, UK; 3 Department of Acute Medicine, Sandwell and West Birmingham NHS Foundation Trust, Birmingham, United Kingdom; 4 South Central Ambulance Service NHS Foundation Trust, Bicester, UK; 5 Hampshire Hospitals NHS Foundation Trust, Winchester, UK; 6 NHS West Hampshire Clinical Commissioning Group, Eastleigh, UK; 7 Nuffield Department of Primary Care Health Sciences, University of Oxford, Oxford, UK

**Keywords:** Early warning score, Continuity of care, Patient safety, Hospital referrals, Secondary care, Retrospective studies, Critical care, Hospitalization, Primary health care

## Abstract

**Background:**

The National Early Warning Score (NEWS) calculated from physiological observations provides a simple away to identify and respond to the deteriorating patient. There is increasing interest in the application of NEWS to facilitate referrals from the community.

**Aim:**

To establish whether elevated NEWS are associated with adverse outcomes at 5 and 30 days when obtained in a community setting at the time of transfer to an acute setting.

**Design & setting:**

A retrospective service evaluation was undertaken using a database of emergency admissions to secondary care from two NHS district general hospitals within the South of England between January 2018 and April 2019.

**Method:**

The performance of NEWS recorded in a community setting to predict death or critical care admission at 5 and 30 days was calculated using established thresholds.

**Results:**

2786 referrals from primary care were analysed. The 5 day and 30 day mortality was 2.2% (1.7 to 2.8) and 7.1% (6.2 to 8.1). The prevalence of the composite outcome was 3.4% (2.8 to 4.2) at 5 days and 8.5% (7.5 to 9.6) at 30 days. The risk of adverse outcomes increased incrementally with increasing NEWS. When calculated at the point of referral from primary care the positive predictive value of death at 5 and 30 days was 15% (95% confidence intervals [CI] = 12 to 19) and 23% (95% CI = 17 to 30) in the high-risk NEWS group.

**Conclusion:**

Elevated NEWS obtained in the community during the process of emergency admission are associated with adverse outcomes. Communicating NEWS may allow downstream care to be better calibrated to risk.

## How this fits in

Transfer of care from the community setting to hospital represents a critical interface in the acute care pathway. There has been a growing call for NEWS to be incorporated into the referral process, but the utility of NEWS in this role has not been established in clinical studies. These findings demonstrate the ability of the first NEWS value obtained in the community to predict death or need for intensive care admission. This suggests NEWS could play a useful role in acute referrals from the community by alerting the receiving team to heightened risk and thereby allowing care to be tailored accordingly.

## Introduction

The number of emergency admissions to hospitals has increased by 40% over the last decade.^1^ Optimising the delivery of acute care to meet these challenges is a strategic priority for healthcare organisations.^2^ The process of managing acutely unwell patients is often complex, commonly requiring multiple transfers of care between clinical teams both across the community–hospital interface and within hospitals. Communication using structured handover is recognised as a key component of quality in emergency and acute medical care.^3^ Conveying the severity of physiological disturbance to the downstream care provider is a key element of safe and effective handover.^4^

Early warning scores (EWS) are calculated from routine physiological observations to provide a single aggregated value representing the risk of future deterioration. EWS have been shown to predict a range of clinical outcomes including death, cardiac arrest, and critical care admission within the inpatient population.^5–7^ NHS England currently mandates the use of National Early Warning Score 2 (NEWS2), a specific EWS, in all acute hospital and ambulance trusts but use in primary care is not compulsory.^8^ NEWS2 has been advocated as a tool to facilitate planning, preparation, and prioritisation when acute illness requires escalation of care from the community to the hospital, but concerns have been raised regarding the absence of validation studies in the primary care population.^9,10^ NICE recommends that people with suspected sepsis in the community have a complete set of observations recorded but does not currently support converting the observations to a NEWS to guide care, due to an absence of evidence in support of this approach.^11,12^ A better understanding of the prognostic value of NEWS obtained in the primary care setting is required before widespread implementation can be advocated with confidence.

This study was undertaken to determine the association between a single NEWS obtained in the community and adverse outcomes in a population of patients deemed to require secondary care assessment following contact with a primary care clinician.

## Methodology

### Evaluation setting

A retrospective service evaluation of consecutive referrals to two district general hospitals within Hampshire Hospitals NHS Foundation Trust was undertaken to determine the association between NEWS recorded in a community setting following assessment by a primary care clinician. A trust-wide database was established in January 2018 to record the details of all patients referred acutely to medical and surgical specialities. Data from January 2018 to April 2019 were analysed.

NEWS comprises seven physiological variables: systolic blood pressure, heart rate, respiratory rate, body temperature, oxygen saturation, use of any supplemental oxygen, and level of consciousness. The score is summed to calculate an aggregate score between 0 and 20, which is used to trigger a standardised escalation policy.^13^ Scores ≤4 are considered low-risk, 5–6 are medium-risk, and scores of ≥7 are high-risk.

The database recorded NEWS scores from two separate sources. The first source consisted of NEWS recorded by the admitting team during the referral process. Physiological observations were obtained by the referring clinician and converted to a NEWS by the admitting clinician. In some cases a NEWS was calculated by the referring clinician directly and communicated to the admitting clinician. These two possibilities were not differentiated within the dataset. A second source of NEWS was provided by the South Central Ambulance Service. The first NEWS recorded by ambulance in all patients conveyed to hospital by ambulance was linked directly to the hospital database. The authors assessed NEWS using death and the composite outcome of death or critical care admission at 5 and 30 days.

This study specifically examined NEWS as opposed to NEWS2, an updated version of NEWS endorsed by NHS England and NHS Improvement as a universal EWS.^8^ NEWS2 is expected to replace NEWS overtime, but few providers had made the transition at the time of this evaluation.^14^ While the revised procedure for aggregating scores in NEWS2 may lead to different absolute mortalities within each risk group, the general association between score and outcome is unlikely to be affected significantly.

### Statistical analysis

Descriptive data are presented as mean and standard deviation (SD), or median and interquartile range (IQR) for non-normally distributed data. Differences in NEWS between groups were compared using the Mann-Whitney U test. NEWS risk thresholds ≥5 and ≥7 as defined by the Royal College of Physicians escalation policy were used to calculate standard statistical measures of diagnostic performance. Receiver operated characteristics (ROC) curves were calculated and the associated area under the curve (AUC) reported. Optimal cut-points were established by maximising the Youden index. The relationship between adverse outcome and NEWS controlling for age was assessed within a multiple logistic regression model. Proportions are presented with 95% CIs. The odds ratio of death between those with NEWS recorded on referral from primary care and those without were used to estimate the impact of confounding by indication. Statistical analysis was performed using the R statistical package (version 2.13.1www.R-project.org).

## Results

### Baseline characteristics

**Figure 1.**
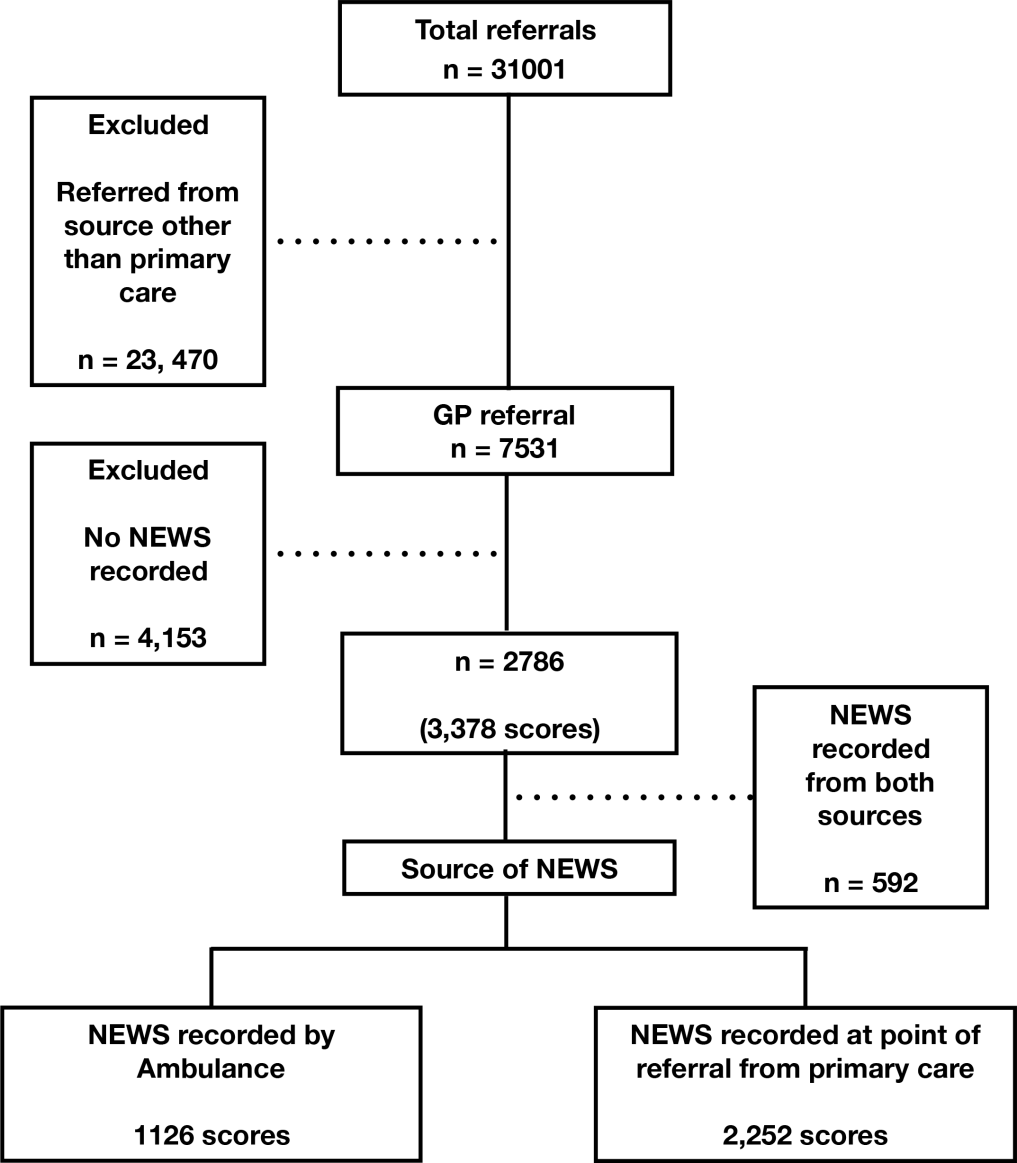
Flow diagram demonstrating study population.

**Figure 2.**
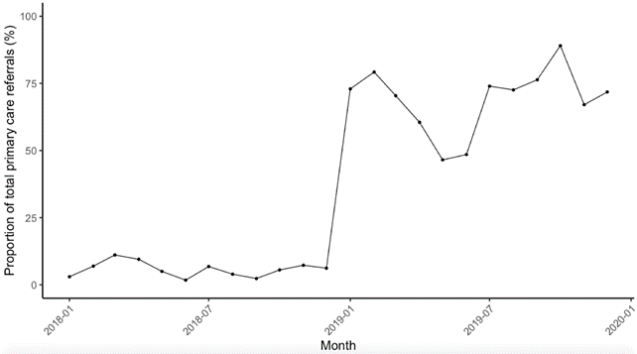
Proportion of total primary care referrals with a NEWS recorded at the point of referral across the evaluation period.

In total, 7531 patients were referred for assessment by a primary care clinician. Within the database, 37.0% (*n* = 2786) referrals had a NEWS recorded. NEWS were recorded by the admitting clinician at the point of referral in 33.5% (*n* = 2522); by the ambulance service in 15.0% (*n* = 1126) referrals; and 7.8% (n=592) referrals had a NEWS recorded initially at the point of referral and then subsequently by the ambulance on conveyance to hospital (Figure 1). Referrals to the medical team represented 96.1% (*n* = 2678) of recorded episodes and referrals to the surgical team 3.9% (*n* = 108). The median monthly proportion of patients with a NEWS recorded at the point of referral was 30.6% (range 1.7% to 89.0%). The proportion of referrals with a recorded NEWS increased progressively over the evaluation period (Figure 2). A large increase in the proportion of referrals with a NEWS recorded occurred in December 2018, coinciding with the implementation of a trust-wide policy mandating the use of NEWS for this purpose.

The proportion of patients with NEWS recorded on referral in the low-risk group (NEWS ≤4) was 33.1% (*n* = 1848), 10.2% (*n* = 230) in the medium-risk group (NEWS 5 or 6), and 7.7% (*n* = 174) in the high-risk group (NEWS ≥7). The comparative values in NEWS recorded by the ambulance were 66.2% (*n* = 745) in the low-risk group (NEWS ≤4), 17.7% (*n* = 199) in the medium-risk group (NEWS 5 or 6), and 16.2% (*n* = 182) in the high-risk group (NEWS ≥7).

The characteristics of the cohort and prevalence of comorbidity stratified by source of NEWS is shown in Table 1. The 5-day mortality was 2.2% (1.7 to 2.8) and the prevalence of the composite outcome of death or critical care admission was 3.4% (2.8 to 4.2). The corresponding prevalence at 30 days was 7.1% (6.2 to 8.1) and 8.5% (7.5 to 9.6). In patients requiring transfer by ambulance, the median NEWS was 3 (IQR 1 to 5). By comparison, the median NEWS recorded by the admitting clinician on referral from primary care was 1 (IQR 0 to 4). This difference was statistically significant (*P*<0.001).

**Table 1.** Demographics of cohort

	**Referrals from primary care**
**Demographic**	**NEWS recorded by ambulance, % (*n*)** **(*n=*** ***1*** ***126)***	**NEWS recorded at point of referral, % (*n*)** **(*n* =** **2252)**
Mean age, years (SD)	76 (15)	65 (21)
Frailty	20.8 (234)	10.6 (239)
Dementia	15.2 (171)	7.5 (169)
ReSPECT form	29.0 (327)	17.9 (403)
Receiving chemotherapy	3.2 (36)	2.5 (56)
Receiving specialist palliative care	13.1 (148)	7.5 (169)
Death at5 days	3.6 (41)	1.9 (43)
Death or critical care at 5 days	5.2 (59)	3.2 (72)
Death at30 days	11.7 (132)	5.8 (131)
Death or critical care at 30 days	13.5 (152)	7.3 (164)

ReSPECT form = Recommended Summary Plan for Emergency Treatment form.

### Diagnostic performance of NEWS

The AUC of NEWS to predict death and the composite outcome of death or critical care admission was 0.70 (0.63 to 0.78) and 0.71 (0.64 to 0.77), respectively, at 5 days; and 0.66 (0.61 to 0.71) and 0.66 (0.62 to 0.71) at 30 days (Table 2). The optimal cut-point was 4 for all outcomes. The corresponding values using NEWS recorded by the ambulance service were 0.74 (0.66 to 0.81) and 0.64 (0.60 to 0.69) at 5 days; and 0.65 (0.60 to 0.70) and 0.64 (0.6 to 0.69) at 30 days. The optimal cut-point was a NEWS value of 4 to identify mortality at 5 days, and death or critical care admission at 30 days; and a NEWS value of 5 to identify mortality at 30 days, and death or critical care admission at 5 days. The association between NEWS and adverse outcomes remained statistically significant (*P*<0.01) for all measured adverse outcomes in a logistic regression model controlled for age.

**Table 2.** Summary of diagnostic performance of NEWS to predict adverse outcome

	**5 day outcome**	**30 day outcome**
	NEWS ≥5	NEWS ≥7	NEWS ≥5	NEWS ≥7
	Death (95% CI)	Death or critical care admission (95% CI)	Death (95% CI)	Death or critical care admission (95% CI)	Death (95% CI)	Death or critical care admission (95% CI)	Death (95% CI)	Death or critical care admission (95% CI)
**NEWS recorded by GP**								
Sensitivity	49%(33-65)	38%(31-47)	21%(10-36)	25%(18-33)	37%(29-46)	37%(30-45)	23%(16-31)	24%(18-32)
Specificity	83%(81-84)	84%(82-85)	93%(91-94)	94%(92-95)	83%(82-85)	84%(82-85)	93%(92-94)	94%(92-95)
Positive predictive value	5%(3-8)	15%(12-19)	5%(2-10)	22%(16-29)	12%(9-16)	15%(12-19)	17%(12-24)	23%(17-30)
Negative predictive value	99%(98-99)	95%(94-96)	98%(98-99)	94%(93-95)	96%(95-96)	94%(93-95)	95%(94-96)	94%(93-95)
Positive likelihood ratio	2.8(2.1–3.9)	2.3(1.9–2.9)	2.8(1.5–5.1)	3.9(2.8–5.3)	2.2(1.8–2.8)	2.3(1.8–2.8)	3.4(2.4–4.8)	3.8(2.8–5.2)
Negative likelihood ratio	0.6(0.5–0.8)	0.7(0.7–0.8)	0.9(0.7–1.0)	0.8(0.7–0.9)	0.8(0.7–0.9)	0.8(0.7–0.9)	0.8(0.8–0.9)	0.8(0.7–0.9)
**NEWS recorded by ambulance**								
Sensitivity	62%(46-77)	53%(45-62)	42%(27-59)	32%(24-40)	54%(45-62)	53%(45-61)	24%(18-30)	31%(24-39)
Specificity	67%(64-70)	69%(66-72)	85%(83-87)	86%(84-88)	69%(66-72)	69%(66-72)	86%(84-88)	86%(84-88)
Positive predictive value	7%(4-10)	21%(17-25)	9%(6-15)	26%(20-33)	19%(15-23)	21%(17-26)	24%(18-30)	26%(20-33)
Negative predictive value	98%(97-99)	91%(88-93)	98%(96-98)	89%(87%–91%)	92%(90-94)	90%(88-92)	86%(84-88)	89%(87-91)
Positive likelihood ratio	1.9(1.5–2.5)	1.7(1.4–2.1)	2.8(1.9–4.1)	2.3(1.7–3.1)	1.7(1.4–2.1)	1.7(1.5–2.1)	1.7(1.3–2.3)	2.2(1.7–3.0)
Negative likelihood ratio	0.6(0.4–0.8)	0.7(0.6–0.8)	0.7(0.5–0.9)	0.8(0.7–0.9)	0.7(0.6–0.8)	0.7(0.6–0.8)	0.9(0.8–1.0)	0.8(0.7-.0.9)

NEWS = national early warning scores.

### Risk of adverse outcomes

**Figure 3.**
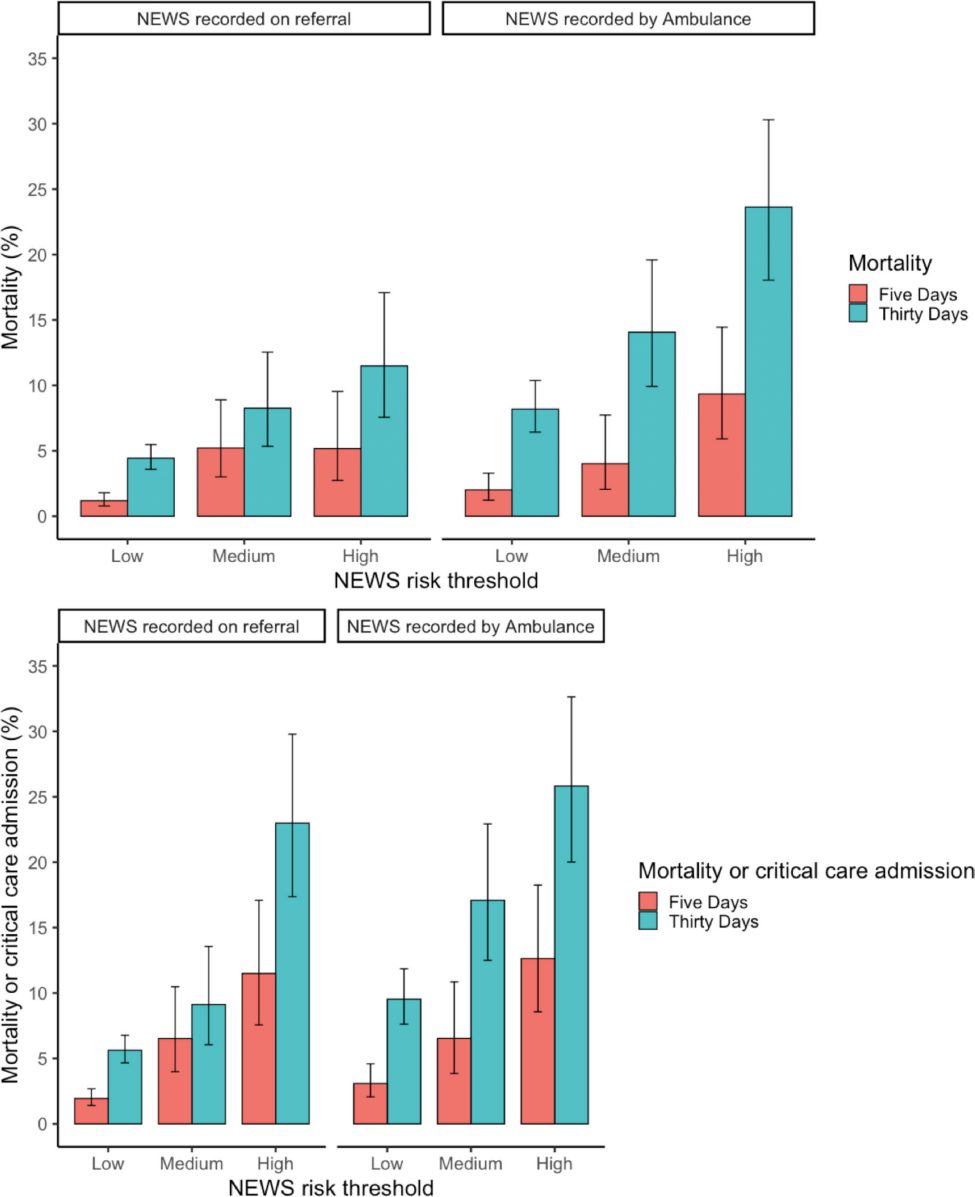
Adverse outcomes stratified by source of NEWS and risk group

Estimates of the absolute risk of death and the composite outcome of death or critical care admission expressed as a proportion at different NEWS thresholds are shown in figure 3. Adverse outcome was more common in medium- and high-risk groups. In the patient group referred from primary care requiring transfer to hospital by ambulance, death or critical care admission occurred in 8.2% (95% CI = 6.4 to 10.4) of patients in the low-risk group at 30 days.

### Estimate of confounding by indication

Comparison was made between groups with a recorded NEWS on referral from primary care and those without scores, to quantify the effect of confounding by indication. The 5 day mortality was 1.9% (95% CI = 1.4 to 2.6) in patients with NEWS recorded and 1.4 (95% CI = 1.1 to 1.7) in patients without. The 30 day mortality was 5.8 (95% CI = 4.9 to 6.9) in patients with NEWS recorded and 6.0 (95% CI = 5.4 to 6.7) in patients without. The odds ratio of death in the patient group with NEWS recorded on referral compared with the patient group without was 1.4 (95% CI = 0.9 to 2.0) at 5 days, and 1.0 (95% CI = 0.97 to 1.18) at 30 days.

## Discussion

This study evaluated the ability of a single NEWS obtained during escalation of care from a community setting to predict adverse outcomes. A clear relationship between elevated scores and mortality at 5 and 30 days was observed. While there is no consensus on the absolute positive predictive value that would encourage use of the score, its relative simplicity makes it a potentially useful and convenient method of communicating risk to the next immediate provider of care in a care chain from community to hospital. Patients assessed in primary care and who require ambulance transfer to facilitate emergency admission to secondary care appear to represent a group at particularly high risk of adverse outcome. This may be explained by the older age and higher prevalence of conditions associated with frailty in this group.

### Strengths and limitations

These findings link NEWS obtained in the community with relevant outcome data within a large contemporary patient cohort. The sample sizes compare favourably with previous studies in this area. Several important limitations should be acknowledged, however, when interpreting these observations.

This evaluation was conducted within a single NHS trust. Only 30.6% of referrals had a NEWS recorded within the database used. The progressively increasing proportion of patients with NEWS recorded with the passage of time suggests the low uptake rate in the initial period of the evaluation represented the prevailing culture of referral at the time, rather than inherent issues with the use of NEWS in primary care. However, even within the context of hospital policy mandating the recording of NEWS on referral, uptake was variable, suggesting not all practitioners recognise or acknowledge the value of NEWS in all circumstances. NEWS may have been more likely to be communicated and recorded in sicker patients. This would be expected to bias the present study’s estimates towards an overestimation of risk. The authors attempted to quantify the effect of this bias by comparing the odds ratio of death between those with and without NEWS recorded. The differences in groups were not statistically different, allowing greater confidence that the estimates of mortality are valid. The majority of patients referred from primary care had low-risk scores. The confidence intervals around estimates in the high-risk group are wide, reflecting the relatively small number of patients within these groups.

The multiple logistic model was controlled for age. Other important covariates, such as validated measures of comorbidity and sex, were not recorded in the database and therefore could not be controlled for in a multiple logistic model.

Analysis was undertaken in a patient group in which the decision to refer the patient for emergency assessment had already been made. It is not possible to know whether the NEWS value directly influenced this decision. Regarding the ambulance derived NEWS, it is not known whether the score was communicated to the ambulance service during the referral process and whether this influenced the response.

Establishing the prognostic significance of changes in NEWS measured at different points may aid better calibration of the acute care pathway to risk. In this evaluation, the heavy skew towards low-risk scores and the fact that the most scores changed little between time points made addressing this important point problematic within the confines of this relatively small sample size.

### Comparison with existing literature

NEWS in this cohort demonstrated moderate discriminatory performance in predicting outcomes at 5 days, and relatively poorer performance at 30 days. Unplanned emergency admissions are unpredictable by nature and are influenced by many factors in addition to the severity of physiological disturbance on admission. Previous studies of NEWS in the inpatient and pre-hospital setting reported superior discriminatory performance than observed in the present evaluation. This may reflect the use of the highest NEWS in a series over time as the predictive variable rather than a single score at a discrete time point, as in the present evaluation.^15,16^

A key unanswered question is whether NEWS adds additional prognostic information to standard clinical assessment. A recent study of an out-of-hours primary care service calculated NEWS retrospectively from physiological variables obtained at the time of assessment.^17^ The individual physiological observations were known to the clinician, but not summarised as an aggregate score. NEWS was a poor predictor of whether an individual patient was subsequently referred acutely to secondary care. The relatively high estimates of mortality observed within the present evaluation raises the possibility that the poor correlation between NEWS and referral may reflect underappreciation of risk rather than a deficiency of NEWS in the primary care setting. An advantage of an aggregated score is its ability to highlight the importance of relatively minor derangement in multiple domains, which can easily be overlooked.

### Implications for research and practice

NEWS should be seen as a simple bedside adjunct to clinical assessment, as evidenced by the small but appreciable risk of adverse outcome in the low-risk group. Some have raised concerns regarding NEWS replacing clinical judgment in primary care, and creating a barrier to admission in patients with low-risk scores.^18^ Although a randomised trial of NEWS would be required confirm its usefulness beyond doubt, given its simplicity, ubiquitous use in other parts of the acute care pathway, and relatively convincing observational data showing the association of elevated NEWS with adverse outcomes, this burden of proof may be deemed unnecessary before more widespread adoption is considered appropriate.

In conclusion, elevated NEWS obtained in the community during the process of emergency admission following assessment by a primary care clinician are associated with increased risk of adverse outcomes at 5 and 30 days. Communicating NEWS to the admitting team when referring from the community may allow subsequent care to be better calibrated to risk from the point of admission.
